# Plasmonic Microcantilever with Remarkably Enhanced Photothermal Responses

**DOI:** 10.1038/s41598-017-05080-y

**Published:** 2017-07-06

**Authors:** Naikun Gao, Dongfang Zhao, Ran Jia, Dongdong Zhang, Duo Liu

**Affiliations:** State Key Laboratory of Crystal Materials, Shandong University, 27 South Shanda Road, Jinan, Shandong 250100 P. R. China

## Abstract

Plasmonic nanostructures exhibit abundant optoelectronic properties. We explore here the technological potentials of plasmonic nanostructures as active component to actuate microcantilever sensors. We find that the photothermal excitation of microcantilevers can be greatly enhanced by Au nanoparticle (NPs). A detailed investigation reveals that the enhancement is wavelength dependent and can be attributed to selective excitation of localized surface plasmon resonance (LSPR). The associated effects are discussed based on a thorough examination of the geometric aspects of Au NPs, microcantilever lengths, and incident optical power. Some technological advantages offered by this method are also discussed.

## Introduction

Microcantilever sensors have proven to be useful tools for highly sensitive detection of small signals^1–4^. A prerequisite for high performance microcantilever sensors lies on effective excitation of the microcantilevers. To date, a variety of excitation methods have been established^5–8^. Among these, photothermal excitation is particularly of interests due to remote and non-contact interaction, characterized by a clean frequency response free of spurious resonances. It is thus considered quite suitable for high speed, high resolution, and quantitative analysis, in particular in liquid or harsh environments where other techniques are not easily applicable^9^. For example, photothermal excitation allows atomic force spectroscopy (AFM) imaging to be remarkably simple, extraordinarily stable, and extremely accurate^10–12^. However, photothermal excitation still suffers from low excitation efficiency^13^. The input optical power must be sufficiently high to effectively actuate a microcantilever, which may lead to undesired side effects such as mechanical nonlinearity, frequency shift, and even optical damages^14^.

Plasmonic nanostructures are abundant in optical properties and can help trap light at metal/dielectric interface^15, 16^. The previous decade has witnessed the development of numerous novel applications related to plasmonic nanostructures in photocatalysis^17^, solar cells^18^, photodetectors^19^, biosensors^20^, light emitting diode (LED)^21^, photothermal therapy^22^, and surface enhanced Raman scattering (SERS)^23^. We explore in this article the feasibility of using plasmonic nanostructures to tune the vibration behaviors of microcantilevers and understand some of the fundamental aspects.

## Results and Discussion

**Figure 1:**
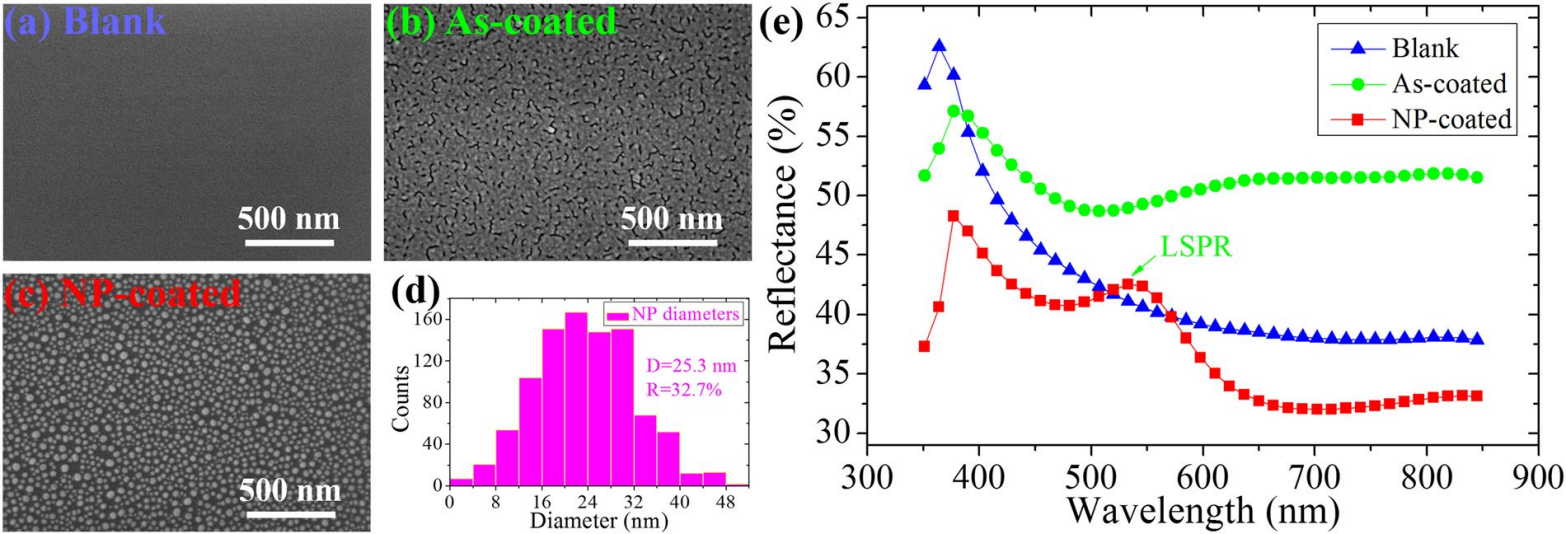
SEM images of the blank (**a**), as-coated (**b**) and NP-coated (**c**) microcantilevers after Au deposition for 120 s, followed by annealing at 500 °C in N_2_ for 1 hour. (**d**) The histogram for the particle size distribution obtained from a statistical analysis of 950 NPs. (**e**) The optical reflectance spectra for the blank, as-coated and NP-coated microcantilevers.

To understand the excitation principle, it is essential to know the surface morphologies and the optical properties of the microcantilevers. Figure 1a–c show the SEM images of the blank, the as-coated, and the NP-coated microcantilevers. The blank microcantilever shows a very smooth surface. The as-coated microcantilever (*T*_*s*_ = 120s) shows continuous Au film characterized by a network of small microcracks with an average length of ~35 nm and width of ~13 nm. Thermal treatment at 500 °C results in the formation of thermodynamically more stable Au NPs, similar to previous investigations^24^. Figure 1d shows the histogram for the particle size distribution obtained from a statistical analysis of 950 NPs. The particle diameters range from several nanometers to ~50 nm and mainly concentrated between 16 nm and 32 nm. A statistical analysis reveals that the average particle size (D) and the surface coverage ratio (R) are ~25.3 nm and ~32.7%, respectively.

Figure 1e shows the reflectance spectra of the three microcantilevers. The blank microcantilever features the reflectance spectrum of silicon^25^. The as-coated microcantilever shows increased reflectance in VIS-IR region (~400–850 nm) due to the presence of Au film^26^. The NP-coated microcantilever shows reduced reflectance characterized by a reflectance peak around 534 nm, which can be attributed to localized surface plasmon resonance (LSPR) of Au NPs, collective resonance of the conduction electrons in response to incident optical waves^27^.

### Wavelength dependence

**Figure 2:**
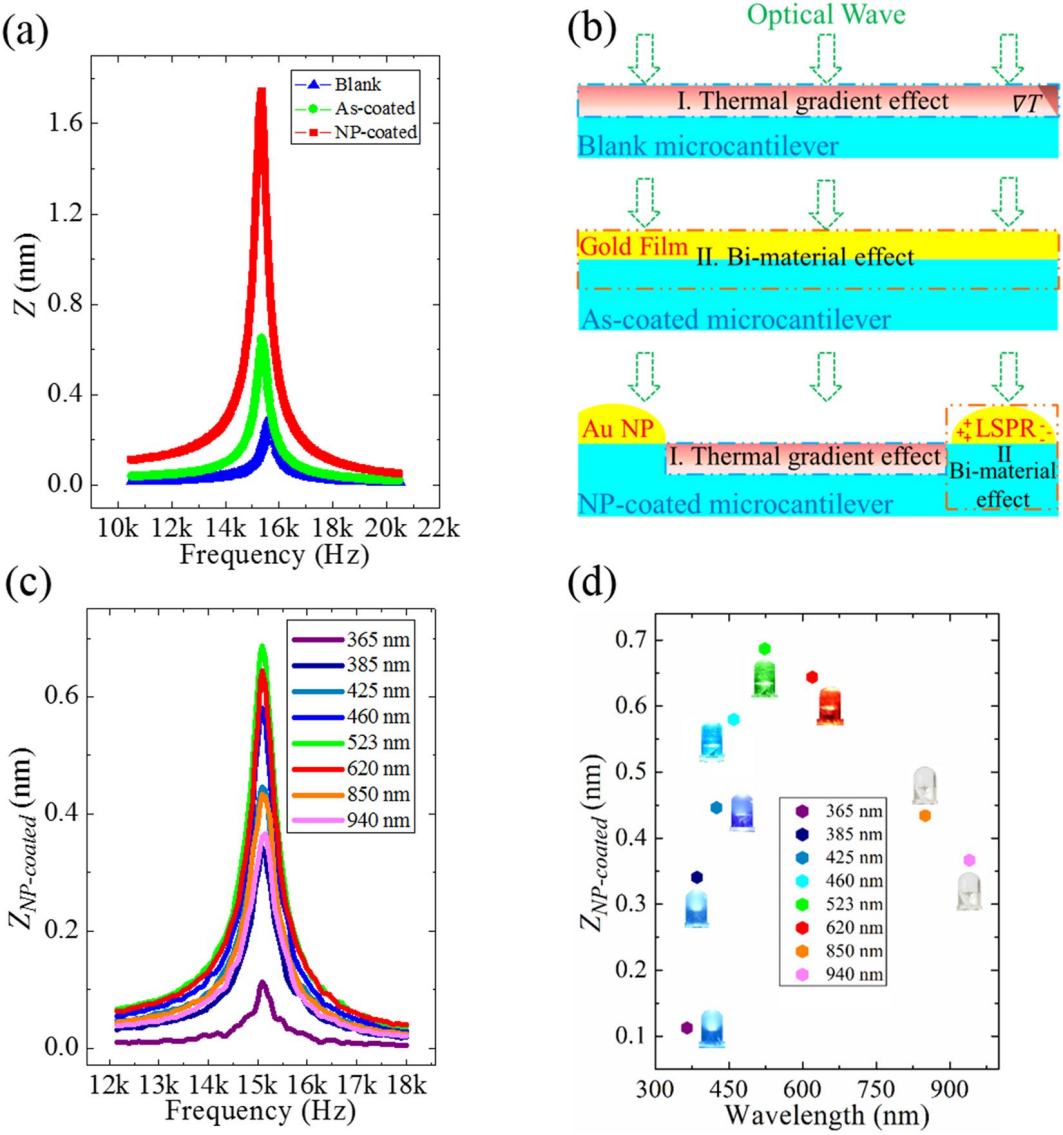
(**a**) The frequency response of the 1st resonant mode for the blank, as-coated and NP-coated microcantilevers (*l*_*Si*_ = 350 µm, *T*_*s*_ = 120 s). (**b**) A schematic illustration of the excitation mechanisms. The Blank microcantilever vibration is dominated by the thermal gradient effect (upper). The As-coated microcantilever vibration is dominated by the bi-material effect (middle). The NP-coated microcantilever vibration contain both the thermal gradient effect for the area without Au NPs and the bi-material effect for the area covered with Au NPs (below). (**c**) The frequency responses of the NP-coated microcantilever under modulated illumination by LEDs of different wavelengths. **(d**) The variation of the vibration amplitudes at resonance as a function of the LED wavelengths.

Au NPs can greatly change the vibration behaviors of a microcantilever. Figure 2a shows the frequency responses of the blank, the as-coated and the NP-coated microcantilevers (*l*_*Si*_ = 350 µm, *T*_*s*_ = 120 s). A LED (523 nm, 35 mW/cm^2^) was used for the excitation of the microcantilevers. It is evident that both the Au film and the Au NPs can greatly increase the vibration amplitude (*Z*).

Photothermal excitation of microcantilever can be attributed to thermal expansion induced by the thermal gradient effect^28^ and/or the bi-material effect (Figure 2b)^29^. The thermal gradient effect originates from the cyclic heat flux along the thickness direction, while the bi-material effect comes from different thermal expansion coefficients of two materials, e.g. Au and Si. As a result, modulated optical illumination excites the microcantilever to vibrate. Notably, the excitation of a microcantilever covered with Au NPs should contain both the thermal gradient effect for the area without Au NPs and the bi-material effect for the area covered with Au NPs (Figure 2b). In our case, our calculation indicates that the bi-material effect is in phase (*α*_*Au*_ = 14.2 µm·m^−1^·K^−1^, *α*_*Si*_ = 2.6 µm·m^−1^·K^−1^), but greater than the thermal gradient effect^10, 30^.

Figure 2c shows the frequency response of the NP-coated microcantilever (*l*_*Si*_ = 350 µm, *T*_*s*_ = 120 s) under modulated illumination by LEDs of different wavelengths, but the same power density (*I* = 15 mW/cm^2^). Each experiment had been repeated three times to ensure stable responses. The maximum vibration amplitudes (*Z*_*NP-coated*_) as a function of wavelengths are summarized in Figure 2d. The vibration amplitude shows a maximum enhancement of ~598% at 523 nm, close to the LSPR of Au NPs.

LSPR depends on the geometric aspects of the metal nanostructures^31^, and surrounding dielectric environment^32^. For Au NPs with diameters well below the wavelength of light, a point dipole model can be used to describe the absorption and scattering of light. The scattering cross-section (*C*_*scat*_) and absorption cross-section (*C*_*abs*_) are given by ref. 33:

1 $${C}_{scat}=\frac{{1}}{{6}\pi }{(\frac{{2}{\pi }}{{\lambda }})}^{{4}}{|\beta |}^{{2}},\,{C}_{abs}=\frac{{2}{\pi }}{{\lambda }}{\rm{Im}}[\beta ]$$

where

2 $$\beta ={3}V[\frac{{\varepsilon }_{{Au}}/{\varepsilon }_{{me}}-{\rm{1}}}{{\varepsilon }_{{Au}}/{\varepsilon }_{{me}}+{\rm{2}}}]$$

where *β* is the polarizability of the Au NP, *V* is the particle volume, *ε*_*Au*_ and *ε*_*me*_ are the dielectric function of Au NP and the embedding medium, respectively. When Re(*ε*_*Au*_/*ε*_*me*_) = −2 is satisfied, a resonant enhancement for both scattering and adsorption can be achieved. As a result, the Au NPs serve as nanoscale antenna to harvest photons, resulting in a significant temperature rise^34^, which benefit both the thermal gradient effect and the bi-material effect.

### Effects of Sputtering Time

**Figure 3:**
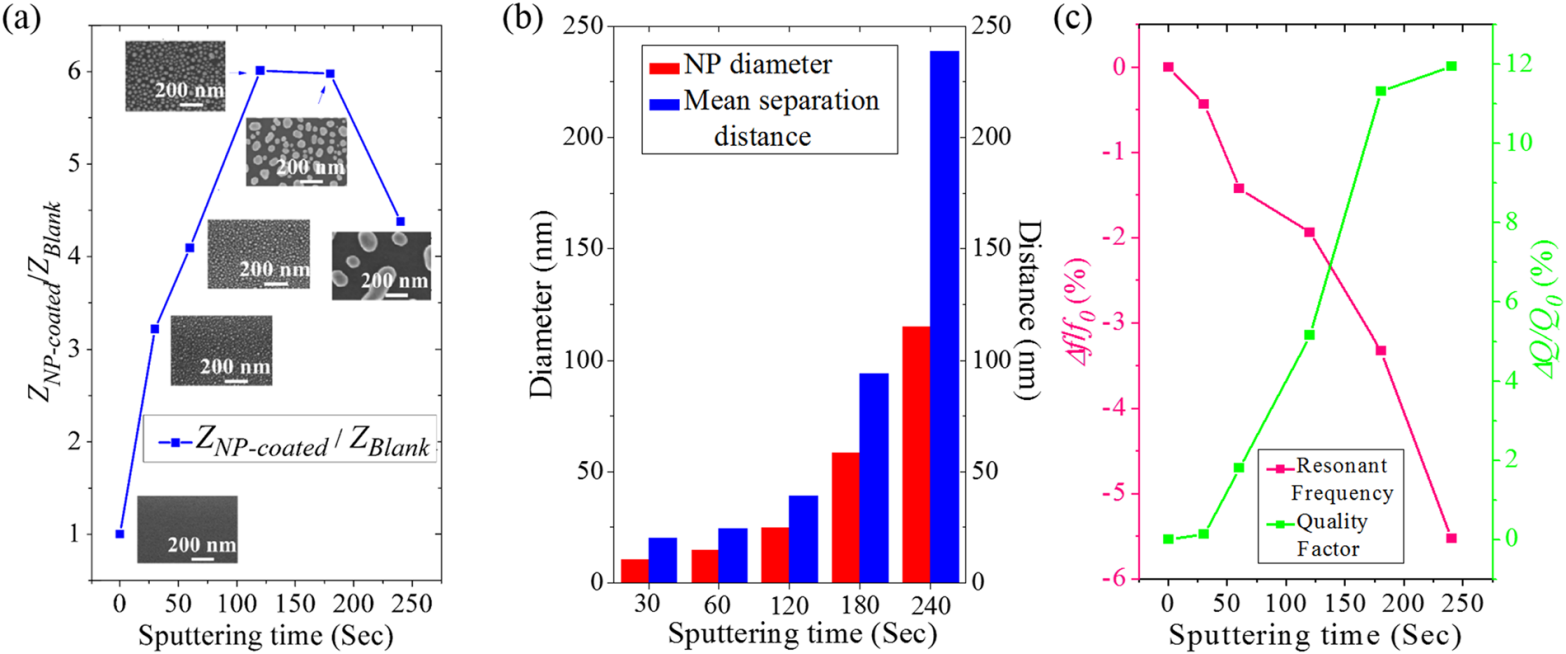
(**a**) Amplitude enhancement for microcantilevers (*l*_*Si*_ = 350 μm) coated with Au NPs obtained under different *T*_*s*_, with SEM images showing the morphological features. (**b**) The NP diameter and interparticle spacing as a function of the sputtering time (**c**) Variation of the resonant frequencies and quality factors of the microcantilevers obtained under different *T*_*s*_.

We then evaluated the effects of the sputtering time (*T*_*s*_) on the vibration behaviors of the microcantilever (*l*_*Si*_ = 350 µm) before and after fabrication of Au NPs. Figure 3a shows the enhancements of the vibration amplitudes (*Z*_*NP-coated*_/*Z*_*Blank*_) at the 1st resonance for microcantilever obtained under different *T*_*s*_ ranging from 0s, 30s, 60s, 120s to 240s. The enhancement increases rapidly and reaches a maximum value (598 %) when *T*_*s*_ = 120 s, then gradually decreases.

The observation can be understood based on (1) the photothermal size effect and (2) the accumulative effect of Au NPs^35^. The photothermal size effect depends on the radius of the NPs^32^. The accumulative effect comes from coupled addition of heat fluxes generated by single NPs in proximity. The total heat generation can be written as a sum over all NPs: *Q*(*r, t*) = *Σ*_*i*_ *Q*_*i*_(*r, t*), where the *Q*_*i*_(*r, t*) describes heat generation by the *i*th NP. The accumulative effect has been experimentally confirmed on Au nanoclusters^36^, Ag nanoclusters^37^, and silica-core/gold-shell nanoclusters^38^. Figure 3b shows a histogram that summarizes the NP diameter and interparticle spacing as a function of the sputtering time, both of which increase with the sputtering time from 30 s to 240 s. A combination of both effects result in maximum amplitude enhancement for the microcantilever obtained at *T*_s_ = 120 s.

Figure 3c shows the variation of the 1st resonant frequencies (*f*_*1st*_) and the quality factors (*Q*) as a function of *T*_*s*_. All the values were extracted from the frequency response curves by Lorentzian fittings. It is found that increasing *T*_*s*_ from 0 s to 240 s will reduce *f*_*1st*_ by 5.5% and increase *Q* by 12%. According to the classical beam theory, the decrease of *f*_*1st*_ can be attributed to the added mass Δ*m* by the Au NPs^39^. Similarly, since $Q=\sqrt{k({m}_{Blank}+{\rm{\Delta }}{m}}/\gamma$, where *m*_*blank*_ is the mass of the blank microcantilever, *k* and *γ* are the spring constant and the damping coefficient, added mass could also increase *Q* ^40^.

### Effects of microcantilever length

**Figure 4:**
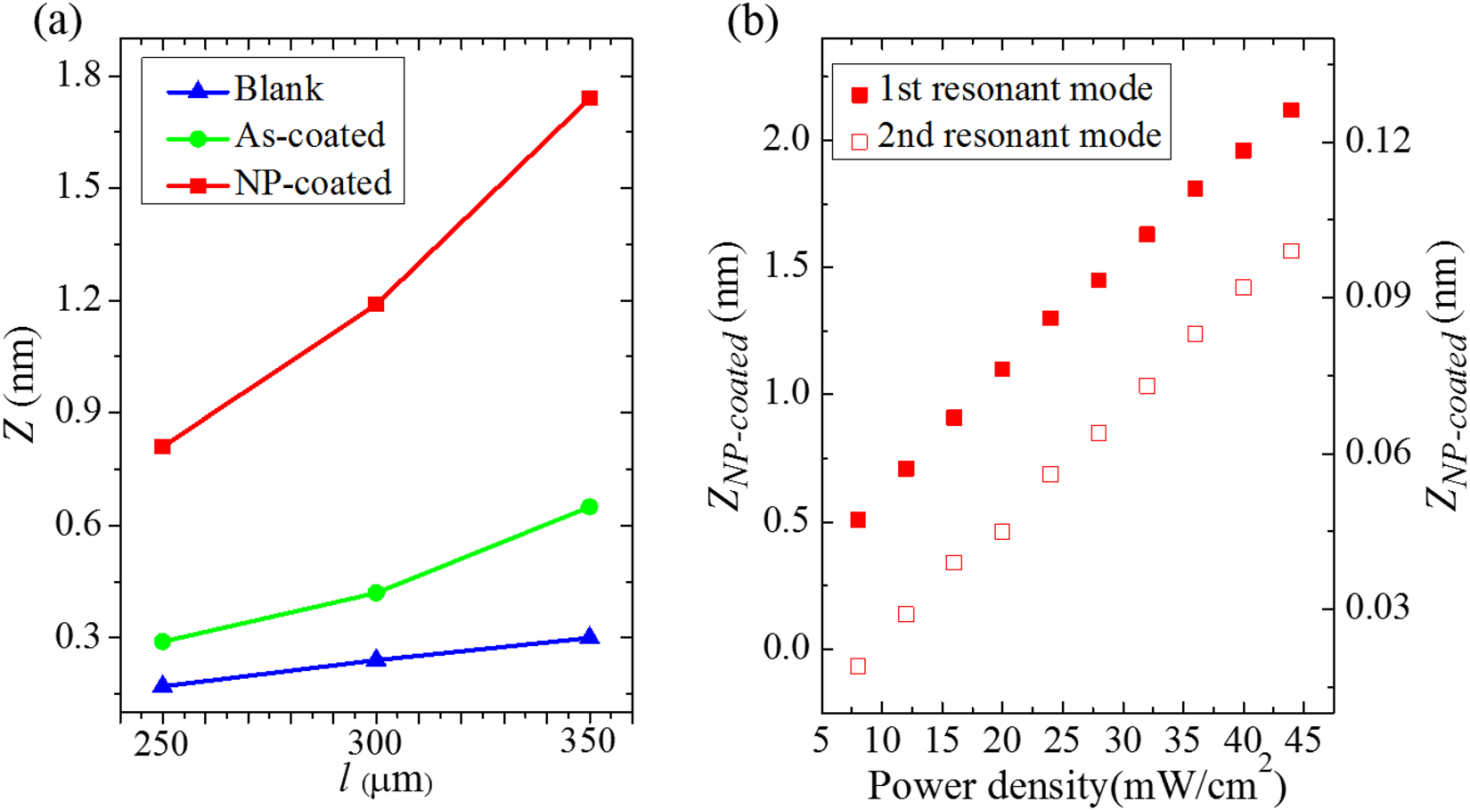
(**a**) Vibration amplitude of the blank, as-coated and NP-coated microcantilevers of different lengths. (**b**) Vibration amplitude of the first two resonant modes of the microcantilever (*l* = 350 μm) as a function of LED power (λ = 523 nm).

We further investigate the effect of microcantilever lengths on the vibration amplitude by using microcantilevers of different lengths (*l*_1_ = 250 μm, *l*_2_ = 300 μm, *l*_3_ = 350 μm) with *T*_*s*_ = 120s. The results are summarized in Figure 4a. It is evident that the longer the microcantilever, the higher the vibration amplitude. The ratios of the vibration amplitude for the microcantilevers of different lengths are 1:1.41:1.76 (Blank), 1:1.45:2.24 (As-coated) and 1:1.47:2.15 (NP-coated), respectively.

Note that the vibration of the blank microcantilevers can be described by the thermal gradient effect with the vibration amplitude *Z*_*Blank*_ given by ref. 28.

3 $${Z}_{Blank}=-{\alpha }_{Si}\frac{{\rm{\Delta }}T}{{t}_{Si}}\frac{{{{l}}_{Si}}^{{2}}}{{2}}={\Gamma }_{{1}}\cdot {{l}}^{{2}}\cdot {\rm{\Delta }}{T}$$

where *α*_*Si*_ is the thermal expansion coefficient of Si, ${\Delta }{T}({t})=({2}{\beta }I\sqrt{{r}})/\sqrt{{\pi }{\kappa }{\rho }C},$ with *I* being the optical power density, *r* the irradiation time*, β*, *κ*, *ρ* and *C* the light absorption, thermal conductivity, density and specific heat of the Si microcantilever, respectively.

In contrast, the vibration of the as-coated microcantilevers can be described by the bi-material effect with the vibration amplitude *Z*_*As-coated*_ given by ref. 41.

4 $${{Z}}_{{As}-{coated}}=\frac{{3}{{l}}^{2}({{\alpha }}_{{Au}}{-}{{\alpha }}_{{Si}})\Delta {T}}{{{t}}_{{Au}}+{{t}}_{{Si}}}{W}={{\Gamma }}_{2}\cdot {{l}}^{2}\cdot {\Delta }{T},$$

with *W* = (1 + *t*_*Au*_/*t*_*Si*_)^2^/[3(1 + *t*_*Au*_/*t*_*Si*_)^2^ + (1 + *E*_*Au*_ *t*_*Au*_/*E*_*Si*_ *tSi*)$({t}_{Au}^{2}/{t}_{Si}^{2}+{E}_{Si}{t}_{Si}/{E}_{Au}{t}_{Au})$], where *t*_*Au*_ is the thickness of the gold film, *α*_*Au*_ and *E*_*Au*_ are the thermal expansion coefficient and Young’s moduli of Au.

Both effects show that the vibration amplitude is proportional to the square of the microcantilever lengths, which yields a ratio of 1:1.44:1.96 for microcantilevers with lengths *l*_*1*_ = 250 nm, *l*_*2*_ = 300nm, *l*_*3*_ = 350 nm, which agrees well with the experimental values.

Notably, the vibration amplitudes of the NP-coated microcantilevers are much greater than the blank and as-coated microcantilevers. This observation suggests that Au NPs are more efficient for photothermal excitation. We believe it can be ascribed to the accumulative thermal effect of Au NPs, which hinder the heat diffusion on the microcantilevers.

Furthermore, we measure the vibration amplitude of both the 1st and 2nd resonant modes of the NP-coated microcantilever (*l*_*Si*_ = 350 μm, *T*_*s*_ = 120 s) upon optical excitation by LEDs of different *I* (523 nm), as shown in Figure 4b. The vibration amplitude increases linearly with the power density from 8 mW/cm^2^ to 44 mW/cm^2^. The good linearity indicates that the NP-coated microcantilever can serve as an excellent optical power meter. The detection limit of optical power meter can be determined by the slope of the ratio of microcantilever amplitude and LED power, and exhibit a detection limit of 0.37 pm/nW and 0.018 pm/nW for the 1st and 2nd resonant modes, respectively.

## Conclusions

In summary, we develop a method to improve the photothermal excitation efficiency of microcantilevers by using Au NPs. Our results show that Au NPs can greatly increase the vibration amplitude by 598% than the blank one in response to 523 nm light. It is confirmed that the enhancement arises from LSPR and depends on the geometric aspects of Au NPs and the microcantilever length. The Au NPs can also increase the quality factor of the microcantilevers due to effects of added mass. We believe that the accumulative thermal effect of Au NPs can benefit the photothermal conversion efficiency. This plasmonic microcantilever has significant technical implications and can serve as an excellent optical power meter with a detection limit of 0.37 pm/nW.

## Methods

Au NPs were fabricated on silicon (Si) microcantilevers (MikroMasch, length *l*_*Si*_ = 350 μm, 300 μm, 250 μm, width *w*_*Si*_ = 35 μm, thickness *t*_*Si*_ = 1 μm) by a sputtering post annealing technique. To achieve this, Au film was first sputtering coated onto the microcantilevers in a sputter coating system (ETD 2000, China), at 10 mA in vacuum for different periods of times (*T*_*s*_) ranging from 30s, 60s, 90s, 120s to 240s, respectively. The microcantilevers were then annealed at 500 °C in nitrogen for 1 hour to obtain Au NPs of different geometric aspects.

**Figure 5:**
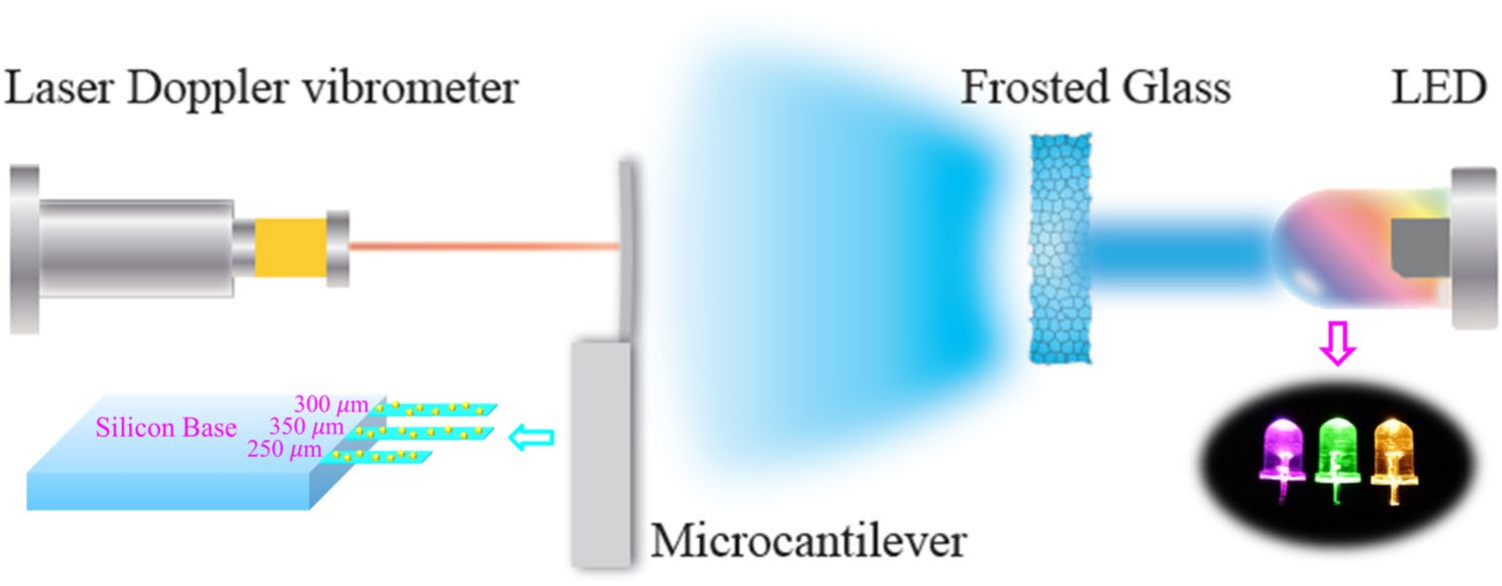
Schematic diagram of the experimental setup used for photothermal excitation of a microcantielver by using LED.

Figure 5 shows the experimental setup used for the measurement of the vibration behaviors of the microcantilevers. We use light emitting diode (LED) as the excitation source. LED offers several advantages over laser diode for microcantilever excitation that include: (1) lower cost and longer service time (>100 k hours), (2) wider spectral coverage, (3) better modulation flexibility, and (4) better system integrability with microcantilever. In our experiments, we picked up LEDs commercially available in market with central emission wavelengths at 365 nm, 385 nm, 425 nm, 460 nm, 523 nm, 620 nm, 850 nm and 940 nm, respectively. The LEDs were modulated by an arbitrary waveform generator (Model 33220a, Agilent, USA). The emission profile of the LEDs was adjusted by a frosted glass to achieve homogeneous light distribution on the microcantilevers. The power density (*I*) of the light was calibrated by an optical power meter (PD300-UV-193 ROHS, OPHIR, Israel). The microcantilever vibration was monitored by a laser Doppler vibrometer (OFV-5000/534, Polytec, Germany), equipped with a lock-in amplifier (Model 7265, Signal recovery, USA). The experimental data were collected by a data acquisition card (Model PCI-6111, NI, USA) and processed by a PC^42^.

## References

[1] Dohn S, Hansen O, Boisen A (2006). Cantilever based mass sensor with hard contact readout. Appl. Phys. Lett..

[2] Sahin O, Magonov S, Su C, Quate CF, Solgaard O (2007). An atomic force microscope tip designed to measure time-varying nanomechanical forces. Nat. Nanotechnol..

[3] Shibata M, Uchihashi T, Ando T, Yasuda R (2015). Long-tip high-speed atomic force microscopy for nanometer-scale imaging in live cells. Sci. Rep..

[4] Yim C (2015). CO2-Selective Nanoporous Metal-Organic Framework Microcantilevers. Sci. Rep..

[5] Huefner M, Pivonka A, Kim J, Ye C, Blood-Forsythe MA, Zech M, Hoffman JE (2012). Microcantilever Q control via capacitive coupling. Appl. Phys. Lett..

[6] Gaillard J, Skove M, Ciocan R, Rao AM (2006). Electrical detection of oscillations in microcantilevers and nanocantilevers. Rev. Sci. Instrum..

[7] Torras N, Zinoviev KE, Marshall JE, Terentjev EM, Esteve J (2011). Bending kinetics of a photo-actuating nematic elastomer cantilever. J. Appl. Phys. Lett..

[8] Feng Z (2012). X. Resonant actuation of microcantilever by pulse wave of one-nth the resonant Frequency. Appl. Phys. Lett..

[9] Nishida S (2008). Photothermal excitation and laser Doppler velocimetry of higher cantilever vibration modes for dynamic atomic force microscopy in liquid. Rev sci instrum..

[10] Labuda A (2014). Photothermal excitation for improved cantilever drive performance in tapping mode atomic force microscopy. Microscopy and Analysis..

[11] Kocun M, Labuda A, Gannepalli A, Proksch R (2015). Contact resonance atomic force microscopy imaging in air and water using photothermal excitation. Rev sci instrum..

[12] Yamashita H, Kodera N, Miyagi A (2007). Tip-sample distance control using photothermal actuation of a small cantilever for high-speed atomic force microscopy. Rev sci instrum..

[13] Nishida S, Kawakatsu H, Nishimori Y (2009). Photothermal excitation of a single-crystalline silicon cantilever for higher vibration modes in liquid. J. Vac. Sci. Technol. B..

[14] Yildiz F, Gulsoy M, Cilesiz I (2016). An experimental study on photothermal damage to tissue: the role of irradiance and wavelength. Laser Phys..

[15] Jain PK, Huang X, El-Sayed IH, El-Sayed MA (2008). El-Sayed. Noble Metals on the Nanoscale: Optical and Photothermal Properties and Some Applications in Imaging, Sensing, Biology, and Medicine. Acc. Chem. Res..

[16] Hutter E, Fendler JH (2004). Exploitation of Localized Surface Plasmon Resonance. Adv. Mater..

[17] Zhou X, Liu G, Yu J, Fan W (2012). Surface plasmon resonance-mediated photocatalysis by noble metal-based composites under visible light. J. Mater. Chem..

[18] Zhang Q (2015). Plasmonic GaInP solar cells with improved energy conversion efficiency. RSC Adv..

[19] Luo LB (2014). Light trapping and surface plasmon enhanced high-performance NIR photodetector. Sci. Rep..

[20] Wang TJ, Lin WS (2006). Electro-optically modulated localized surface plasmon resonance biosensors with gold nanoparticles. Appl. Phys. Lett..

[21] Sung JH (2010). Enhancement of electroluminescence in GaN-based light-emitting diodes by metallic Nanoparticles. Appl. Phys. Lett..

[22] Huang XH, Jain PK, El-Sayed IH (2008). Plasmonic photothermal therapy (PPTT) using gold nanoparticles. Lasers Med Sci..

[23] Gkogkou D (2016). Polarization- and Wavelength-Dependent Surface-Enhanced Raman Spectroscopy Using Optically Anisotropic Rippled Substrates for Sensing. ACS Sens..

[24] Tesler AB (2011). Rubinstein, I. Tunable Localized Plasmon Transducers Prepared by Thermal Dewetting of Percolated Evaporated Gold Films. J. Phys. Chem. C.

[25] Jannat A, Leea W, Akhtar MS, Li ZY, Yanga OB (2016). Low cost sol–gel derived SiC–SiO_2_ nanocomposite as antireflection layer for enhanced performance of crystalline silicon solar cells. Appl surf sci..

[26] Rohsenow W. M. & Choi, H. Y. *In Heat Mass and Momentum Transfer* (Prentice Hall, New York, 1961).

[27] Luk’yanchuk BS, Tribel’skiĭ MI, Ternovskiĭ VV (2006). Light scattering at nanoparticles close to plasmon resonance frequencies. J. Opt. Technol..

[28] Marti O (1992). Mechanical and thermal effects of laser irradiation on force microscope cantilevers. Ultramicroscopy..

[29] Salazar A, Sánchez-Lavega A, Terrón JM (1998). Effective thermal diffusivity of layered materials measured by modulated photothermal techniques. J appl phys..

[30] Kiracofe D, Kobayashi K, Labuda A, Raman A, Yamada H (2011). High efficiency laser photothermal excitation of microcantilever vibrations in air and liquids. Rev sci instrum..

[31] Fan X, Zheng W, Singh DJ (2014). Light scattering and surface plasmons on small spherical particles. LIGHT-SCI APPL..

[32] Setoura K, Okada Y, Werner D, Hashimoto S (2013). Observation of Nanoscale Cooling Effects by Substrates and the Surrounding Media for Single Gold Nanoparticles under CW-Laser Illumination. ACS Nano..

[33] Bohren, C. F. & Huffman, D. R. *Absorption and scattering of light by small particles* (Wiley-Interscience, New York; doi:10.1002/9783527618156, 1983).

[34] Keblinski P, Cahill DG, Bodapati A, Sullivan CR, Taton TA (2006). Limits of localized heating by electromagnetically excited nanoparticles. J. Appl. Phys..

[35] Govorov AO, Richardson HH (2007). Generating heat with metal nanoparticles. Nanotoday.

[36] Govorov AO (2006). Gold nanoparticle ensembles as heaters and actuators: melting and collective plasmon resonances. Nanoscale Res Lett.

[37] Jensen TR, Malinsky MD, Haynes CL, Van Duyne RP (2000). Nanosphere Lithography: Tunable Localized Surface Plasmon Resonance Spectra of Silver Nanoparticles. J. Phys. Chem. B..

[38] Hirsch LR (2003). Nanoshell-mediated near-infrared thermal therapy of tumors under magnetic resonance guidance. PNAS..

[39] Gupta A, Akin D, Bashir R (2004). Single virus particle mass detection using microresonators with nanoscale thickness. Appl. Phys. Lett..

[40] Liu Y, Zhao G, Wen L, Xu XZ, Chu J (2011). Mass-loading effect on quality factor of floppy silicon microcantilever in free air space. Micro nano lett..

[41] Timoshenko S (1925). Analysis of Bi-Metal Thermostats. J. Opt. Soc. Am..

[42] Gao NK, Zhao DF, Jia R, Liu D (2016). Microcantilever Actuation by Laser Induced Photoacoustic Waves. Sci. Rep..

